# Postnatal women’s breastfeeding beliefs, practices, and support during the COVID-19 pandemic: a cross-sectional comparative study across five countries

**DOI:** 10.1186/s13006-022-00497-2

**Published:** 2022-08-17

**Authors:** K. P. Coca, E. Y. Lee, L. Y. Chien, A. C. P. Souza, P. Kittikul, S. A. Hong, Y. S. Chang

**Affiliations:** 1 Escola Paulista de Enfermagem, Universidade Federal de São Paulo, São Paulo, Brazil; 2 Department of Nursing, Catholic Kkottongnae University, Cheongju-si, Republic of Korea; 3 Institute of Community Health Care, College of Nursing, National Yang Ming Chiao Tung University, Yang-Ming Campus, Taipei, Taiwan; 4 Breastfeeding Clinic Nakhon Pathom Hospital, Nakhon Pathom, Thailand; 5 ASEAN Institute for Health Development, Mahidol University, Nakhon Pathom, Thailand; 6 Institute for Health and Society, Hanyang University, Seoul, Republic of Korea; 7 Florence Nightingale Faculty of Nursing, Midwifery and Palliative Care, King’s College London, London, UK

**Keywords:** Breastfeeding, Postnatal, COVID-19, Belief, Healthcare support

## Abstract

**Background:**

Women with COVID-19 experienced numerous concerns and doubts about the safety of breastfeeding their babies, and lack of support may have impacted breastfeeding practices. This study aims to compare breastfeeding beliefs, practices, and contact with healthcare professionals regarding the level of postnatal feeding support provided during the COVID-19 pandemic in Brazil, South Korea, Taiwan, Thailand, and the United Kingdom.

**Methods:**

A multi-country cross-sectional study was conducted with postnatal women in five countries. Women up to six months postpartum were invited to complete an online survey concerning the transmission of preventative measures, beliefs toward breastfeeding, infant feeding practices in the last 24 hours and experiences of postnatal infant feeding support between July to November 2021. Bivariate and multivariate analyses were performed to identify the association.

**Results:**

Of the 3,253 eligible responses received, 39.5% of children were aged between one and two months, but in Taiwan (36%) and South Korea (42.8%) they were between three and four months. The mean of the belief score was significantly different among countries (*p* < 0.0001). Women in Brazil and the UK had a higher rate of breastfeeding at the breast (90.7% and 85.4%, respectively) compared to the three Asian countries (*p* < 0.0001) while feeding with expressed breastmilk in Thailand (59.9%), Taiwan (52.6%), and South Korea (50.4%) was higher than the others (*p* < 0.0001). Brazil and UK mothers (mean = 16.0 and 14.5 respectively) had a higher mean score for belief toward breastfeeding during the COVID-19 than the others. These results are inversely associated with breastfeeding but positively related to formula feeding practice. Postnatal feeding support during the COVID-19 pandemic was mainly provided by healthcare professionals (67.1%) and peers / family through face-to-face personal contact (51.6%) in all countries.

**Conclusion:**

Some differences were found in breastfeeding beliefs during the COVID-19 pandemic in Asian countries. A positive breastfeeding belief was associated with the practice of breastfeeding at the breast. Women from all countries received postpartum infant feeding support from health professionals and peers / family through personal contacts. Governments need to emphasize and disseminate the importance of breastfeeding safety, especially in Asian countries.

## Background

The rates of exclusive breastfeeding varied between countries pre-pandemic. For example, at six months postpartum, rates of exclusive breastfeeding were 1% in the UK (2012) [1], 11.4% in South Korea (2011) [2], 14.1% in Thailand (2020) [3], 24.3% in Taiwan (2011) [4], and Brazil 45.8% (2019) [5, 6], for under six months postpartum.

Even in the midst of the COVID-19 pandemic, the rates and number of deaths vary in many countries [7], with breastfeeding being an effective strategy for protecting infants [8]. During the COVID-19 pandemic, women are recommended to breastfeed in the first hour after delivery to enable skin-to-skin contact with their baby, helping them to continue exclusive breastfeeding [7].

Furthermore, women with suspected or confirmed COVID-19 infection are also encouraged to breastfeed [7, 9] since the benefits of breastfeeding outweigh the potential risks of virus transmission [8, 10–12]. In addition, preventative measures should be taken to reduce the transmission from mother to baby, such as washing hands using soap and water before touching the child, expressing breast milk if deciding not to breastfeed directly from the breast, either with a breast pump or hand expression and wearing a mask during breastfeeding [10, 13].

Despite the recommendations, women with COVID-19 reported significant concerns and doubts about the safety of breastfeeding their babies [6]. Furthermore, the number of COVID-19 cases, death rate, and the control measures could impact infant feeding practices through the mother’s perception of breastfeeding against the risk of infection [14]. Nevertheless, an increase in women who avoided using the health system for postpartum care and lactation support and the counseling on offer, tended to feel detached from their babies due to the fear of COVID-19 transmission through breastmilk. This impacted the breastfeeding rates in the short and long term [15, 16]. Therefore, during the COVID-19 pandemic, breastfeeding rates may have been adversely affected by misinformation on the benefits of breastfeeding and infant protection [17]. Furthermore, the provision of breastfeeding support by health services and professionals during the COVID-19 pandemic has been challenging, potentially impacting breastfeeding practice [18, 19].

Since the World Health Organization (WHO) declared COVID-19 a global pandemic, it is important to recognize the impact on women and their breastfeeding practices in different countries. The UK and Brazil experienced higher death rates from the *SARS-CoV-2* infection than Asian countries. There are few studies on the impact of the COVID-19 pandemic on women’s breastfeeding practices among countries [20, 21, 22]. The present study aims to compare breastfeeding beliefs, practices, and level of contact with healthcare professionals for receiving postnatal feeding support during the COVID-19 pandemic in five countries: Brazil, Taiwan, Thailand, South Korea, and the UK, to highlight the similarities and differences internationally.

The acquisition of knowledge concerning the COVID-19 pandemic impact on infant feeding practice and breastfeeding support in countries with various geographical locations and COVID-19 rates may provide valuable insights into breastfeeding promotion during a pandemic like COVID-19. It could contribute to the achievement of several Sustainable Development Goals of the 2030 agenda since breastfeeding should be a priority practice to protect the survival and health of babies and women [23, 24, 25].

## Methods

### Study design and sampling

A multi-country online cross-sectional study was conducted in five countries: Brazil, South Korea, Taiwan (Republic of China), Thailand, and the UK. Postnatal women were invited to participate in a survey concerning their infant feeding practices, experiences of postnatal infant feeding support received, and belief toward transmission and preventative measures for breastfeeding during the COVID-19 pandemic between July 2021 to November 2021.

Women up to six months postpartum, aged between 18 and 49 years (in Taiwan, between 20 and 49 years old), and literate in the country’s official language, were included in the survey. The exclusion criteria consisted of women who were not living in one of the countries under study during the survey period and those who could not read the questions. Since this study used convenience sampling online, no sample size was calculated. Based on the prevalence of infant feeding practices, the sample in each country was considered sufficient for this analysis.

The survey was developed in English and translated into the local languages of participating countries (Portuguese, Korean, Chinese, and Thai), and then back-translated into English. The researchers and some breastfeeding women from each participating country reviewed the content to identify any statements in the research instrument which were unclear, misleading, or highly sensitive and to verify the questions to ensure the validity and reliability of the survey prior to use in an online survey. Based on the results and comments from the data collectors, minor revisions were made to the wording.

### Data collection

Due to the preventative measures imposed to minimize the spread of COVID-19, data were collected using online Google Forms for web-based surveys. The survey information was distributed via e-mail, social media (Facebook, WhatsApp, Instagram, Twitter, etc.), personal networks, groups of health professionals, and not-for-profit organizations. In South Korea, a private company was used for recruitment.

All women who participated voluntarily signed an online informed consent form before starting the survey according to the Ethical Committee approval conditions of each country. In addition, prior to signing the informed consent, information on the study design and purpose were presented, including the assurance of confidentiality.

### Measures of variables

#### The variables investigated included:

*Sociodemographic factors*: maternal age, education level, working status, marital status, residence (urban or rural area), as well as age and sex of the child.

*Infant* feeding practices were assessed with the question: “How was your youngest baby fed in the last 24 hours?”: 1) Breastfeeding (baby only fed directly from the breast); 2) Expressed breast milk; 3) Infant formula; and 4) Solid, semi-solid, or soft foods (including non-breast milk liquids). Participants were also asked: “Have you completely stopped breastfeeding and giving expressed breast milk to your youngest baby?” (stopped breastfeeding, still breastfeeding, never breastfed).

Belief in breastfeeding during the COVID-19 pandemic and concerns about virus transmission through breast milk and preventative measures while breastfeeding were measured using six questions, following WHO recommendations [26]. Women were asked to rate the following statements on a 3-point Likert scale (1=Agree, 2=Uncertain, and 3=Disagree): 1) “COVID-19 can be passed on to the baby through breast milk and breastfeeding”; 2) “If the mother is confirmed or suspected to have the COVID-19 infection, the mother should not breastfeed”; 3) “If the mother is confirmed or suspected to have the COVID-19 infection, the baby should still be immediately placed skin-to-skin and breastfed following delivery”, 4) “If the mother is confirmed or suspected of having the COVID-19 infection, it is safer to give the baby infant formula milk than the mother’s breast milk or practice breastfeeding at the breast”; 5) “A breastfeeding mother who is confirmed or suspected of having the COVID-19 infection should always wear a face mask when breastfeeding”; and 6) “A mother who is confirmed or suspected to have the COVID-19 infection can touch and hold her newborn baby without wearing a face mask”. Statements 3 and 5, which are in favor of breastfeeding [26], were reversely coded before summing. The total score ranged from 6 to 18, with a higher score meaning a more positive belief toward breastfeeding.

*Postpartum infant feeding support* was assessed using three multiple-choice questions: 1) “From whom do you receive postnatal infant feeding support?” (mark all that apply) i) no support received, ii) from healthcare professionals, iii) from spouse / partner, friends, or relative, iv) online support group (e.g., Facebook), and v) other; 2) “How do you make contact with healthcare professionals for postpartum support?” (mark all that apply) i) never, ii) in person, iii) by phone, iv) video, and v) other; and 3) “If you have received breastfeeding support though video contact with any infant feeding supporters (e.g., healthcare professionals, etc.), did you experience any difficulties?” (mark all that apply) i) never had video contact, ii) no difficulty, iii) supporter unable to clearly see the baby latch, iv) supporter was unable to hear me well, v) could not hear the support well, vi) could not see the support well, vii) could not operate the device and breastfeed at the same time, and viii) other.

### Statistical analysis

Descriptive statistics were used to measure the frequency and proportion of categorical variables, such as general characteristics, infant feeding practices, postnatal infant feeding support experience, questions relating to beliefs on breastfeeding during the COVID-19 pandemic, and mean and standard deviation (SD) for continuous variables, such as the total score for beliefs toward breastfeeding and COVID-19. The percentage of variables by country were compared using Chi-square tests or Fisher’s exact test as appropriate for categorical variables. T-tests or one-way analysis of variance (ANOVA) tests were performed to determine the significant associations between the mean belief scores and all examined variables. Bivariate and multivariate analyses of country comparison for infant feeding practice and breastfeeding status (never, stopped breastfeeding, and still breastfeeding) were assessed using binary and multinomial logistic regression, respectively. Associations between infant feeding practices and breastfeeding beliefs were assessed using binary logistic regression. In multivariate analyses, model 1 includes the infant’s age (and country in the total sample), while model 2 also includes the infant’s sex, mother’s age, education, working status, marital status, and type of residence (and country in the total sample) in model 1, with crude and adjusted odds ratios (COR and AOR, respectively) and 95% confidence intervals (CIs) presented. All analyses were conducted using SAS 9.3 (SAS Institute Inc., Cary, NC, USA).

## Results

A total of 3,507 women completed the survey, and 3,253 met the inclusion criteria (Brazil: 560; Taiwan: 614; Thailand: 840; South Korea: 381; the UK: 858). Most of the women were aged between 30 and 39 years (61.7%), 75.8% of whom had a university or a postgraduate degree, 59.2% were on maternity leave, married (95.5%), and lived in an urban area (72.6%). Although 39.5% of children in the study were aged between one and two months, most of those in Taiwan (36%) and South Korea (42.8%) were between three and four months. The sex of the children was similar between boys and girls (Table 1).

**Table 1:** Women’s sociodemographic backgrounds in Brazil, Taiwan, Thailand, South Korea, and the UK

Participants	Total n (%) *N*= 3253	Brazil n (%) *N*= 560	Taiwan n (%) *N*= 614	Thailand n (%) *N*= 840	South Korea n (%) *N*= 381	UK n (%) *N*=858	*p*-value
Maternal age (years)							
18–29	1094 (33.6)	164 (29.3)	204 (33.2)	489 (58.2)	51 (13.4)	186 (21.7)	< 0.0001
30–39	2005 (61.7)	360 (64.3)	397 (64.7)	318 (37.9)	311 (81.6)	619 (72.1)	
41–49	154 (4.7)	36 (6.4)	13 (2.1)	33 (3.9)	19 (5)	53 (6.2)	
Education level							
Secondary or lower	787 (24.2)	85 (15.2)	35 (5.7)	458 (54.5)	34 (8.9)	175 (20.4)	< 0.0001
^a^University / postgraduate	2465 (75.8)	475 (84.8)	579 (94.3)	382 (45.5)	347 (91.1)	682 (79.6)	
Working status							
Yes	564 (17.3)	87 (15.6)	28 (4.6)	357 (42.5)	61 (16)	31 (3.6)	< 0.0001
No	762 (23.5)	104 (18.6)	99 (16.1)	312 (37.1)	197 (51.7)	50 (5.8)	
^b^On maternity leave	1926 (59.2)	368 (65.8)	487 (79.3)	171 (20.4)	123 (32.3)	777 (90.6)	
Marital status (married)	3105 (95.5)	521 (93)	605 (98.5)	759 (90.4)	380 (99.7)	840 (97.9)	< 0.0001
^c^Urban residence	2360 (72.6)	542 (97.1)	535 (87.1)	382 (45.5)	360 (94.5)	541 (63.1)	< 0.0001
Age of child							
1–2 months	1285 (39.5)	238 (42.5)	197 (32.1)	477 (56.8)	73 (19.2)	300 (35)	< 0.0001
3–4 months	1099 (33.8)	195 (34.8)	221 (36)	218 (26)	163 (42.8)	302 (35.2)	
5–6 months	869 (26.7)	127 (22.7)	196 (31.9)	145 (17.2)	145 (38)	256 (29.8)	
Sex of child (boy)	1644 (50.5)	293 (52.3)	316 (51.5)	435 (51.8)	174 (45.7)	426 (49.7)	0.255

^a^The number of missing values is 1 in total and 1 in the UK

^b^The number of missing values is 1 in total and 1 in Brazil

^c^The number of missing values is 2 in total and 2 in Brazil

Missing values were excluded (not counted) in both the descriptive statistics and Chi-squared tests

Table 2 demonstrates the infant feeding practice in the 24 hours prior to the survey, with 73.5% of mothers reporting breastfeeding directly from the breast, 38.3% used expressed breast milk, 40.6% formula milk, 11.9% solid, semi-solid, or soft foods, 11.9%. By country, women in Brazil and the UK had a higher rate of breastfeeding at the breast (90.7% and 85.4%, respectively) compared to the three countries in Asia (*p* < 0.0001), while feeding with expressed breastmilk in Thailand (59.9%), Taiwan (52.6%), and South Korea (50.4%) was higher than in the other countries (*p* < 0.0001). Higher rates were found in Taiwan for feeding with formula milk, and solid, semi-solid, or soft foods, (73.3% and 21.7%, respectively) and South Korea (57.5% and 15.8%) compared to the others. In all countries, 19.3% of mothers reported that they had completely stopped breastfeeding, although the figure was higher in South Korea (33.9%), Thailand (28.2%), and Taiwan (26.6%) than in Brazil and the UK (*p* < 0.0001).

**Table 2:** Infant feeding practices in Brazil, Taiwan, Thailand, South Korea, and the UK

	Total n (%) *N*= 3,253	Brazil n (%) *N*= 560	Taiwan n (%) *N*= 614	Thailand n (%) *N*= 840	South Korea n (%) *N*= 381	UK n (%) *N*= 858	*p*-value
^a^Infant feeding							
BF at breast	2392 (73.5)	508 (90.7)	333 (54.2)	544 (64.8)	274 (71.9)	733 (85.4)	< 0.0001
Breast milk expressed	1246 (38.3)	77 (13.8)	323 (52.6)	503 (59.9)	192 (50.4)	151 (17.6)	< 0.0001
Infant formula	1321 (40.6)	111 (19.8)	450 (73.3)	328 (39.1)	219 (57.5)	213 (24.8)	< 0.0001
Solid, semi-solid, or soft foods	388 (11.9)	39 (7.0)	133 (21.7)	66 (7.9)	60 (15.8)	90 (10.5)	< 0.0001
Have you completely stopped breastfeeding?							
Stopped BF	626 (19.3)	14 (2.5)	163 (26.6)	237 (28.2)	129 (33.9)	83 (9.7)	< 0.0001
^b^Continuing to BF	2493 (76.8)	528 (95.7)	442 (72)	534 (63.6)	239 (62.7)	750 (87.4)	
^c^Never BF	126 (3.9)	10 (1.8)	9 (1.5)	69 (8.2)	13 (3.4)	25 (2.9)	

*BF* breastfeeding

^a^Infant feeding practice in the 24 hours prior to the survey

^b^Breastfeeding includes expressed breast milk

^c^The number of missing values is 8 in the total sample and 8 in Brazil. Missing values were (not counted) excluded in both descriptive statistics and chi-squared tests.

Since the differences in characteristics such as infant’s age between countries may contribute to the varying rates of infant feeding practice, the multivariate associations after adjustment for covariates were examined. The country comparisons of infant feeding practice presented in Table 2 remained similar even after adjustment for covariates in Tables 3 and 4. Mothers from South Korea and Taiwan were associated with higher rates of solid, semi-solid, or soft foods (Table 3), while those in South Korea, Taiwan, and Thailand stopped breastfeeding earlier compared to those from Brazil who had never breastfed (Table 4).

**Table 3:** Bivariate and multivariate analyses of country comparison for infant feeding practice

	Simple			Model 1			Model 2		
	COR	(95% CI)		AOR	(95% CI)		AOR	(95% CI)	
BF at breast									
Brazil	1			1			1		
Taiwan	0.12	(0.09,	0.17)	0.13	(0.09,	0.17)	0.13	(0.09,	0.18)
Thailand	0.19	(0.14,	0.26)	0.18	(0.13,	0.25)	0.17	(0.12,	0.24)
South Korea	0.26	(0.18,	0.38)	0.28	(0.20,	0.40)	0.25	(0.17,	0.36)
UK	0.60	(0.43,	0.85)	0.62	(0.44,	0.87)	0.64	(0.44,	0.91)
Breast milk expressed									
Brazil	1			1			1		
Taiwan	6.96	(5.22,	9.28)	7.34	(5.49,	9.81)	7.45	(5.54,	10.01)
Thailand	9.36	(7.09,	12.35)	9.12	(6.90,	12.04)	12.91	(9.31,	17.89)
South Korea	6.37	(4.66,	8.71)	6.97	(5.07,	9.58)	8.17	(5.85,	11.41)
UK	1.34	(0.99,	1.80)	1.38	(1.03,	1.86)	1.53	(1.12,	2.09)
Infant formula									
Brazil	1			1			1		
Taiwan	11.10	(8.44,	14.60)	11.05	(8.40,	14.55)	11.22	(8.48,	14.86)
Thailand	2.59	(2.02,	3.33)	2.59	(2.01,	3.32)	3.01	(2.24,	4.04)
South Korea	5.47	(4.09,	7.31)	5.45	(4.06,	7.30)	6.69	(4.90,	9.13)
UK	1.34	(1.03,	1.73)	1.33	(1.03,	1.72)	1.34	(1.01,	1.76)
Solid, semi-solid, or soft foods									
Brazil	1			1			1		
Taiwan	3.69	(2.53,	5.39)	3.71	(2.43,	5.66)	5.08	(3.20,	8.07)
Thailand	1.14	(0.76,	1.72)	1.52	(0.97,	2.39)	0.92	(0.55,	1.54)
South Korea	2.50	(1.63,	3.83)	1.73	(1.08,	2.77)	2.25	(1.36,	3.71)
UK	1.57	(1.06,	2.32)	1.29	(0.84,	1.98)	1.51	(0.94,	2.44)

*AOR* Adjusted Odds Ratios, *COR* Crude Odds Ratios

Model 1: Adjusted for infant’s age

Model 2: Adjusted for additional variables, such as maternal age, education, working status, marital status, residence, and infant’s sex in Model 1

**Table 4:** Bivariate and multivariable analyses of the country comparison for breastfeeding status

	Have you completely stopped breastfeeding?																	
	Simple						Model 1						Model 2					
	Stopped BF vs. Never BF			Still BF vs. Never BF			Stopped BF vs. Never BF			Still BF vs. Never BF			Stopped BF vs. Never BF			Still BF vs. Never BF		
	COR	(95% CI)		COR	(95% CI)		AOR	(95% CI)		AOR	(95% CI)		AOR	(95% CI)		AOR	(95% CI)	
Country																		
Brazil	1			1			1			1			1			1		
Taiwan	12.94	(4.51,	37.08)	0.93	(0.38,	2.31)	12.32	(4.29,	35.35)	0.94	(0.38,	2.33)	11.14	(3.83,	32.40)	0.80	(0.32,	2.03)
Thailand	2.45	(1.04,	5.77)	0.15	(0.08,	0.29)	2.76	(1.17,	6.51)	0.14	(0.07,	0.28)	4.23	(1.67,	10.71)	0.28	(0.13,	0.60)
South Korea	7.09	(2.63,	19.11)	0.35	(0.15,	0.81)	6.35	(2.34,	17.18)	0.36	(0.15,	0.83)	6.44	(2.31,	17.95)	0.28	(0.12,	0.66)
UK	2.37	(0.94,	5.99)	0.57	(0.27,	1.19)	2.25	(0.89,	5.70)	0.57	(0.27,	1.21)	2.08	(0.79,	5.48)	0.54	(0.24,	1.19)

*AOR* Adjusted Odds Ratios, *COR* Crude Odds Ratios Model 1: Adjusted for infant’s age

Model 2: Adjusted for additional variables, such as maternal age, education, working status, marital status, residence, and infant’s sex in Model 1

**Fig. 1:**
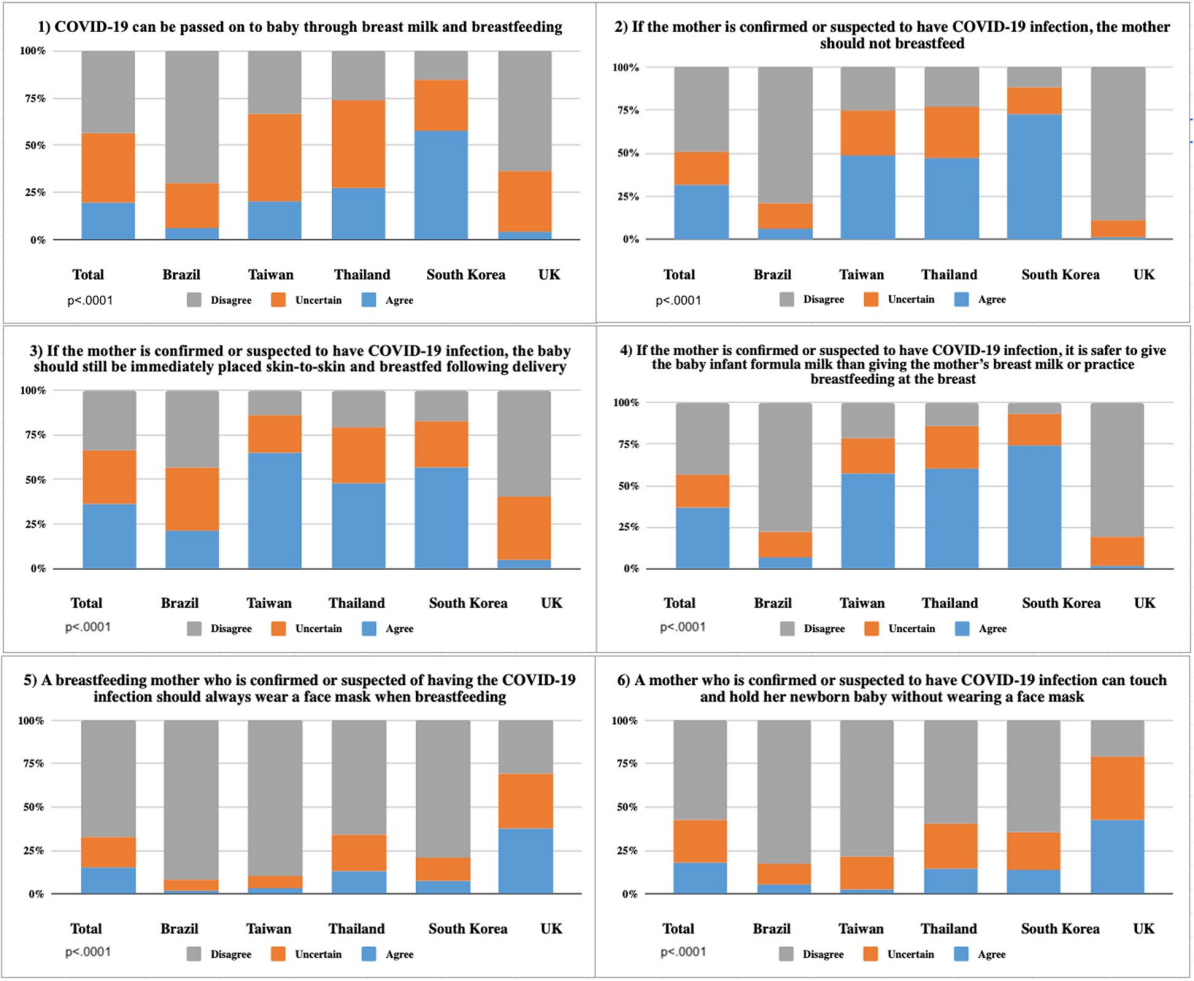
Belief toward breastfeeding during the COVID-19 pandemic by mothers in Brazil, Taiwan, Thailand, South Korea, and the UK

Fig. 1 presents the results for beliefs toward breastfeeding in relation to COVID-19 transmission and preventative measures by country. The belief that the “Coronavirus can pass on to babies through breast milk and breastfeeding” (Question 1) and “When the mother is confirmed or suspected of having COVID-19, she should not breastfeed” (Question 2) were considered to be held by most women from South Korea (57% and 72%, respectively) followed by Thailand (27% and 47%) and Taiwan (20% and 49% respectively). Brazil (6% in both) and the UK (4% and 1%, respectively) presented the lowest rates of agreement with these statements. Similar results were identified in the responses to Question 4: “If the mother is confirmed or suspected of having the COVID-19 infection, is it safer to give the baby infant formula milk than the mother’s breast milk or breastfeeding at the breast?” (Question 4): agreement was 74% in South Korea, 60% in Taiwan, 57% in Thailand, 7% in Brazil, and 2% in the UK.

In response to Question 3: “If a mother is confirmed or suspected of having the COVID-19 infection, should a baby still be immediately placed skin-to-skin and breastfed following delivery?” the disagreement was high in the UK and Brazil (60% and 43% respectively), and lower in the Asian countries: 21% in Thailand, 17% in South Korea, and 14% in Taiwan (Fig. 1).

Mothers were asked about wearing a mask during breastfeeding (Question 5) or taking care of their babies (Question 6) if a breastfeeding mother is confirmed or suspected of having the COVID-19 infection. Women in all countries disagree with both (67% and 57%, respectively), especially those from Brazil (92% and 83%, respectively), followed by Taiwan (90% and 78%), South Korea (79% and 65%), Thailand (65% and 60%), and the UK (31% and 21%) (Fig. 1).

Table 5 shows the mean score for belief toward breastfeeding in relation to COVID-19 transmission and preventative measures by infant feeding practice and country. The mean score for belief toward breastfeeding during the COVID-19 pandemic was 13.3 (SD = 2.7) in the pooled sample. The mean of the belief score was significantly different among countries (*p* <.0001). Brazil and UK mothers (16.0, SD = 2.1 and 14.5, SD = 1.9, respectively) had a higher mean score than the others (12.6 and SD = 2.2 in Taiwan, 12.0 and SD = 2.3 in Thailand, and 11.1 and SD = 1.9 in South Korea). Women breastfeeding at the breast exhibited a high score for beliefs toward breastfeeding in the pooled samples (*p* < 0.0001) and by country, with a statistically significant difference observed in Taiwan (*p* < 0.0001) and the UK (*p* < 0.0001). Meanwhile, those feeding with infant formula had a low score for belief toward breastfeeding in the pooled samples (*p* < 0.0001), and the result was statistically significant in Taiwan, Thailand, and the UK (*p* < 0.0001). Those feeding expressed breast milk and solid, semi-solid, or soft foods had a lower score for belief in the pooled samples (*p* < 0.0001 and *p* < 0.015, respectively), but no significant associations were found at the country level. In addition, the multivariable associations between breastfeeding belief and infant feeding practice (Table 6) indicated a positive association between belief toward breastfeeding at the breast, while the inverse association with infant formula remained significant after adjusting for covariates in models 1 and 2 for the total sample. By country, Taiwan and the UK showed a similarly significant association between breastfeeding at the breast and infant formula, while Thailand and Brazil had an inverse association with infant formula (Table 6).

**Table 5:** Associations with infant feeding practice and breastfeeding belief (score) in Brazil, Taiwan, Thailand, South Korea, and the UK

	Total *N*= 3,253 Mean (SD)	*p*-value	Brazil *N*= 560 Mean (SD)	*p*-value	Taiwan *N*= 614 Mean (SD)	*p*-value	Thailand *N*= 840 Mean (SD)	*p*-value	South Korea *N*= 381 Mean (SD)	*p*-value	UK *N*= 858 Mean (SD)	*p*-value
BF Belief (scores)	13.3 (2,7)		16.0 (2.1)		12.6 (2.2)		12.0 (2.3)		11.1 (1.9)		14.5 (1.9)	< 0.0001
Infant feeding practice												
BF at breast												
Yes No	13.7 (2.7) 12.4 (2.4)	<.0001	16.0 (2.0) 15.5 (2.3)	0.065	13.0 (2.2) 12.3 (2.1)	0.000	12.0 (2.2) 12.0 (2.3)	0.914	11.1 (1.9) 11.1 (1.9)	0.759	14.7 (1.8) 13.5 (2.2)	< 0.0001
Breast milk expressed												
Yes No	12.5 (2.5) 13.9 (2.6)	<.0001	15.7 (2.2) 16.0 (2.1)	0.226	12.7 (2.2) 12.6 (2.2)	0.805	12.0 (2.3) 12.0 (2.3)	0.761	11.0 (1.8) 11.3 (1.9)	0.168	14.6 (2.0) 14.5 (1.9)	0.455
Infant formula												
Yes No	12.5 (2.5) 14.0 (2.6)	<.0001	15.6 (2.3) 16.1 (2.0)	0.083	12.4 (2.1) 13.4 (2.3)	<.0001	11.6 (2.4) 12.2 (2.3)	<.0001	11.0 (1.8) 11.2 (1.9)	0.300	13.9 (2.2) 14.7 (1.7)	< 0.0001
Solid, semi-solid, or soft foods												
Yes No	13.1 (2.5) 13.4 (2.7)	0.015	15.9 (2.2) 16.0 (2.1)	0.826	13.0 (2.1) 12.6 (2.2)	0.117	11.8 (2.0) 12.0 (2.3)	0.500	10.8 (1.6) 11.2 (1.9)	0.100	14.5 (1.9) 14.5 (1.9)	0.919

*BF* breastfed / breastfeeding

**Table 6:** Logistic regression results of infant feeding practice and belief toward breastfeeding (score) in the total sample and by country

		Simple			Model 1			Model 2		
Dependent variable	Independent variable	COR	(95% CI)		AOR	95% CI		AOR	(95% CI)	
Total										
Breastfeeding at breast	BF belief	1.22	(1.18,	1.26)	1.11	(1.07,	1.15)	1.13	(1.09,	1.18)
Expressed breast milk	BF belief	0.82	(0.80,	0.85)	0.99	(0.95,	1.03)	0.98	(0.94,	1.02)
Infant formula	BF belief	0.80	(0.78,	0.83)	0.86	(0.83,	0.90)	0.85	(0.82,	0.89)
Solid, semi-solid, or soft foods	BF belief	0.95	(0.91,	0.99)	1.00	(0.95,	1.05)	0.96	(0.91,	1.02)
Brazil										
Breastfeeding at breast	BF belief	1.124	(0.99,	1.27)	1.11	(0.98,	1.26)	1.12	(0.98,	1.29)
Expressed breast milk	BF belief	0.934	(0.84,	1.04)	0.94	(0.84,	1.05)	0.92	(0.81,	1.03)
Infant formula	BF belief	0.912	(0.83,	1.00)	0.92	(0.84,	1.02)	0.89	(0.81,	0.99)
Solid, semi-solid, or soft foods	BF belief	0.983	(0.84,	1.15)	0.95	(0.79,	1.13)	1.02	(0.82,	1.26)
Taiwan										
Breastfeeding at breast	BF belief	1.16	(1.07,	1.25)	1.17	(1.09,	1.27)	1.16	(1.08,	1.26)
Expressed breast milk	BF belief	1.01	(0.94,	1.09)	1.02	(0.95,	1.11)	1.00	(0.93,	1.09)
Infant formula	BF belief	0.82	(0.76,	0.89)	0.82	(0.76,	0.89)	0.81	(0.75,	0.89)
Solid, semi-solid, or soft foods	BF belief	1.072	(0.98,	1.17)	1.04	(0.90,	1.19)	1.04	(0.90,	1.20)
Thailand										
Breastfeeding at breast	BF belief	1.00	(0.94,	1.07)	1.02	(0.96,	1.08)	1.07	(1.00,	1.14)
Expressed breast milk	BF belief	0.99	(0.93,	1.05)	0.99	(0.93,	1.05)	0.96	(0.90,	1.02)
Infant formula	BF belief	0.89	(0.83,	0.94)	0.88	(0.83,	0.94)	0.86	(0.80,	0.92)
Solid, semi-solid, or soft foods	BF belief	0.96	(0.86,	1.08)	0.91	(0.82,	1.03)	0.97	(0.86,	1.10)
South Korea										
Breastfeeding at breast	BF belief	1.02	(0.90,	1.15)	1.02	(0.90,	1.15)	1.01	(0.90,	1.15)
Expressed breast milk	BF belief	0.93	(0.83,	1.03)	0.93	(0.83,	1.03)	0.92	(0.82,	1.02)
Infant formula	BF belief	0.94	(0.85,	1.05)	0.93	(0.84,	1.04)	0.93	(0.83,	1.04)
Solid, semi-solid, or soft foods	BF belief	0.87	(0.74,	1.03)	0.85	(0.72,	1.01)	0.86	(0.73,	1.02)
UK										
Breastfeeding at breast	BF belief	1.42	(1.28,	1.59)	1.43	(1.28,	1.59)	1.39	(1.24,	1.55)
Expressed breast milk	BF belief	1.05	(0.94,	1.14)	1.04	(0.95,	1.14)	1.04	(0.95,	1.15)
Infant formula	BF belief	0.79	(0.72,	0.86)	0.79	(0.72,	0.86)	0.79	(0.73,	0.87)
Solid, semi-solid, or soft foods	BF belief	0.99	(0.89,	1.11)	0.96	(0.84,	1.10)	0.99	(0.86,	1.13)

*AOR* Adjusted Odds Ratios, *COR* Crude Odds Ratios

Model 1: Adjusted for age of child (and country in the total sample)

Model 2: Adjusted for additional variables, such as maternal age, education, working status, marital status, residence, and infant’s sex in Model 1

Table 7 shows the level of postnatal infant feeding support. The pooled sample shows that postnatal infant feeding support was mainly received from health professionals (67.1%) and the community (spouse / partner / relatives / friends) (51.6%). The two groups provided the predominant support in all countries, while support from health professionals was disproportionately high in Thailand (86.3%) and Taiwan (71.0%). One-third of mothers reported that they had obtained support from online groups, mostly in the UK and Thailand (48.6% and 35.5%, respectively).

**Table 7:** Mothers’ experiences of postnatal infant feeding support received in Brazil, Taiwan, Thailand, South Korea, and the UK

	Total n (%) *N*= 3,253	Brazil n (%) *N*= 560	Taiwan n (%) *N*= 614	Thailand n (%) *N*= 840	South Korea n (%) *N*= 381	UK n (%) *N*= 858	*p*-value
Support for postnatal infant feeding							
No support received	505 (15.5)	83 (14.8)	34 (5.5)	5 (6.6)	137 (36)	196 (22.8)	< 0.0001
Healthcare professional	2182 (67.1)	352 (62.9)	436 (71)	725 (86.3)	179 (47)	490 (57.1)	< 0.0001
Spouse / partner, relative, or friend	1678 (51.6)	359 (64.1)	462 (75.2)	307 (36.6)	108 (28.4)	442 (51.5)	< 0.0001
^a^Online group support	998 (30.7)	110 (19.6)	109 (17.8)	298 (35.5)	64 (16.8)	417 (48.6)	< 0.0001
^b^Other	235 (7.2)	4 (0.7)	154 (25.1)	0	0	77 (9)	< 0.0001
^c^Contact with any infant feeding supporters							
Never	868 (26.7)	135 (24.1)	164 (26.7)	96 (11.4)	180 (47.2)	293 (34.2)	< 0.0001
In person	1812 (55.7)	383 (68.4)	400 (65.2)	485 (57.7)	184 (48.3)	360 (42)	< 0.0001
By telephone	1066 (32.8)	135 (24.1)	114 (18.6)	435 (51.8)	32 (8.4)	350 (40.8)	< 0.0001
Video	285 (8.8)	77 (13.8)	28 (4.6)	72 (8.6)	6 (1.6)	102 (11.9)	< 0.0001
Other	59 (1.8)	16 (2.9)	0	0	0	43 (5)	< 0.0001
Support through video contact with any infant feeding support							
No video contact	*n* = 2968	*n* = 483	*n* = 586	*n* = 768	*n* = 375	*n* = 756	
Never had	2435 (82)	410 (84.9)	486 (82.9)	511 (66.5)	313 (83.5)	715 (94.6)	< 0.0001
Had video contact	*n* = 285	*n* = 77	*n* = 28	*n* = 72	*n* = 6	*n* = 102	
No difficulty	169 (59.3)	57 (74)	22 (78.6)	42 (58.3)	4 (66.7)	44 (43.1)	0.0002
Supporter unable to clearly see the baby’s latch	67 (23.5)	7 (9.1)	4 (14.3)	8 (11.1)	1 (16.7)	47 (46.1)	< 0.0001
Supporter was unable to hear me well	13 (4.6)	1 (1.3)	0	5 (6.9)	0	7 (6.9)	0.2464^d^
Could not *hear* support well	14 (4.9)	2 (2.6)	0	4 (5.6)	1 (16.7)	7 (6.9)	0.2478^d^
Could not *see* support well	12 (4.2)	1 (1.3)	0	4 (5.6)	0	7 (6.9)	0.3215^d^
Could not operate the device and BF at the same time	38 (13.3)	5 (6.5)	1 (3.6)	8 (11.1)	1 (16.7)	23 (22.6)	0.0099
Other	9 (3.2)	2 (2.6)	0	0	0	7 (6.9)	0.1196

*BF* breastfeeding

^a^ Facebook or other group support

^b^Internet and hotline service were mentioned

^c^Most common contact

^d^*p*-value from Fisher’s exact test

Support received in person was the most common in the pooled sample (55.7%) and all countries (ranging from 68.4% in Brazil to 42.0% in the UK). Support by phone was also high in the pooled sample (32.8%), ranging from 51.8% in Thailand and 40.8% in the UK to 8.4% in South Korea. Support via video was reported by 8.8% of women in the pooled sample, with the highest rate being in Brazil (13.8%), followed by the UK (11.9%). Most women receiving support via online video platforms reported no difficulties (59.3%), but when difficulties were experienced, the most common one was that the supporter was unable to clearly see their baby latching on (23.5%), especially in the UK (46.1%). Meanwhile, the figures for women receiving no support were relatively high (26.7%), ranging from 47.2% in South Korea and 34.2% in the UK to 11.4% in Thailand (Table 4).

## Discussion

To our knowledge, this is the first study to examine breastfeeding beliefs, practices, and postnatal infant feeding support during the COVID-19 pandemic across five countries. This multi-country study revealed some differences in beliefs toward breastfeeding during the COVID-19 pandemic in Asian countries compared to Brazil and the UK. Compared to women in the UK and Brazil, a higher proportion of women in Asian countries believed that those suspected or infected with COVID-19 could transmit the virus during breastfeeding through breastmilk and skin-to-skin contact. Compared to the other counties, women in Brazil presented the lowest rate of belief that a face mask should always be worn when breastfeeding and touching and holding the baby. Postnatal women’s beliefs toward breastfeeding may affect breastfeeding practice. Women breastfeeding at the breast had a high score of belief toward breastfeeding, while those feeding with infant formula had a lower score. Women reported that postpartum infant feeding support was received mostly from health professionals and peers / family through personal contact in all countries, while the support via online groups was also relatively higher in Thailand and the UK compared to the remaining countries. More than 10% of women in Brazil and the UK reported receiving lactation support via video contact.

### Belief toward breastfeeding

In this study, the mean scores for belief toward breastfeeding in relation to COVID-19 transmission and preventative measures through infant feeding practices were significantly different among countries. A high rate of belief was expressed by the participants when asked about the “transmission of COVID-19 through breastmilk” (Question 1) and “Should women avoid breastfeeding if they are suspected of being infected with COVID-19?” (Question 2) were found in three Asian countries. One reason for this could be that information regarding the safety of breastfeeding during the COVID-19 pandemic was not widely disseminated in Asian countries compared to the UK and Brazil, despite recommendations from several international health agencies and medical societies. Furthermore, during the early stages of the COVID-19 pandemic, health professionals such as the American Academy of Pediatrics [27] and other associations [28] suggested that babies be temporarily separated from their mothers after birth and recommended that breast milk be expressed as a precautionary measure due to concerns about the risk of COVID-19 transmission through breastfeeding.

As soon as evidence was available that transmission of SARS-CoV-2 via breast milk was unlikely [29] and that the impact of breastfeeding would guarantee food safety for children [30], the recommendation to breastfeed grew, with most agencies and medical societies becoming unified in their views [31].

The participants expressed a low rate of belief when asked about “skin-to-skin and breastfeeding following delivery” (Question 3) in all three Asian countries compared to the others. Different postpartum practices in hospitals and maternity services which did not follow the WHO recommendations caused confusion among women about whether or not they should breastfeed their babies [32].

Regarding the practice of wearing a facemask when holding the baby, including during feeding (Questions 5 and 6), the findings reveal that fewer mothers in Brazil were in agreement with this practice during breastfeeding, touching, and holding their newborn baby. Despite the mothers being recommended to protect their nose and mouth with a mask during breastfeeding [10], they appeared to be less concerned about the transmission of COVID-19, preferring to focus on the importance of face-to-face interaction with the baby for brain development [33], nurturing, and bonding to forge a deep shared connection [34].

### Breastfeeding practice

Global breastfeeding rates are generally low [8]. The results of this study reveal that 73% of the women in all the countries under study breastfed at the breast in the 24 hours preceding the survey. Brazil presented the highest breastfeeding rates at 91%, followed by the UK (85%) and South Korea (72%). Due to the difference in COVID-19 pandemic waves between countries, it has proven difficult to compare our results with other studies on the impact of breastfeeding. Despite the global breastfeeding rates being generally low, in the countries we analyzed [1–6] , the rates were higher. A study carried out in April 2020 during a UK lockdown period identified that infant feeding was influenced by the mother’s negative emotions and anxiety when they had more than one child to take care of [35]. Around 27% of women faced barriers to continuing with breastfeeding due to the pandemic lockdown [36]. Another study in the UK, from May 27 to June 2020, showed that 59% of women who delivered during lockdown exclusively breastfed / mixed-fed their infants compared to 39% who delivered before the COVID-19 pandemic [37]. In Thailand, from July — October 2020 and December 2020 to February 2021, after the lockdown from April — June the same year, a slight decrease of 4.3% was exhibited in breastfeeding practice during the COVID-19 lockdown [38]. Furthermore, a study in Italy from March — May 2020 showed similar results, with a decrease in women exclusively breastfeeding compared to before the COVID-19 pandemic (2018) [39], and this was also the case in the United States of America for women who gave birth before 2020 [19]. Despite these findings, a study in China carried out from August to October 2020 to compare the infant feeding experiences of women who delivered before and during the COVID-19 pandemic in Beijing identified that breastfeeding practice rates were maintained during the pandemic [20].

Despite the benefits of breastfeeding and the recommendations made during the COVID-19 pandemic [10], concerns and fears about the infection being transmitted from mother to infant through breastfeeding could have affected infant feeding practice. Furthermore, mothers may have been influenced by various factors when deciding how to feed their infant since breastfeeding media and beliefs during the COVID-19 pandemic varied according to the government regulations, policies, socioeconomic status, and health inequity in each country [40]. Thus, further studies are needed to identify how breastfeeding beliefs toward COVID-19 transmission and prevention measures affect breastfeeding practices in various settings and populations. This study found that a positive breastfeeding belief was associated with the baby being breastfed at the breast and inversely associated with infant formula. All three Asian countries presented lower rates of breastfeeding at the breast and exhibited lower scores for belief compared to Brazil (16) and the UK (14.5), which both showed higher rates of breastfeeding at the breast. Asian women also have a similar or slightly lower rate of breastfeeding using expressed breast milk, while the UK and Brazil had a higher rate of breastfeeding at the breast. We found that 60% of women in Thailand express their breast milk, followed by 53% in Taiwan and 50% in South Korea.

Several factors may interfere with breastfeeding practice, such as educational level [41] and delivery experience [20]. Expressing breast milk may encourage continued breastfeeding [10, 23]. A study of Singaporean Chinese women showed the increased practice of expressed milk and combination feeding, defined as breast milk and non-breast milk fed via bottle and breast, while direct feeding at the breast showed a decreasing trend over time [42]. Women tended to express their breast milk when they did not want to breastfeed in public or had returned to work [23]. An increasing number of working mothers in Asia are changing their infant feeding practices due to increased participation in the labor force among Asian women (Thailand 59.2% and South Korea 53.2%) [43]. During the COVID-19 pandemic, women were more likely to have struggled with increased childcare demands [14]. Breastfeeding at the breast in public places or at work remains challenging for mothers, especially in Asia. In Korea, women who return to work after maternity leave stated that their work status directly affected their decision not to breastfeed. The primary reason given for not breastfeeding was that “it is not easy to express milk at work.” Furthermore, the expressed breastfeeding rate was higher than breastfeeding at the breast: breastfeeding mostly with an occasional bottle with expressed breast milk (44.2%) vs. breastfeeding only (26.9%) [44]. The women’s characteristics in this study showed little difference in work status, and no studies are currently available for comparison. Therefore, further research should be conduted to identify any differences.

Another study showed the influence of social policy on breastfeeding duration, such as the breastfeeding policy of the hospital and national parental leave, although social policy was not found to be statistically associated with the breastfeeding duration in a recent Korean study [45]. Furthermore, although women with a higher education level tend to know about the health benefits of breastmilk, they are more likely to be employed or involved in out-of-home activities. Thus, it is often difficult for women to feed their babies directly and instead use expressed breastmilk or combination feeding. In comparing the results, the participants in this study were found to have a high educational level (75.8% had a university or postgraduate degree), but 59.2% were on maternity leave. Infant feeding formula was used by 41% of women in the pooled sample, with the rate being higher in Taiwan at 73%, followed by South Korea at 57%. A high formula feeding rate was exhibited in Asian countries during the COVID-19 pandemic. Despite the benefits of expressed breast milk to support continued breastfeeding practice, women who exclusively expressed in early postpartum may not achieve long-term breastfeeding [46]. Using breast milk instead of formula feeding gives babies the benefits of human milk but can also reduce the practice of breastfeeding at the breast and result in increased formula feeding [47].

### Postnatal feeding support

Face-to-face support for breastfeeding by professionals and / or peers improved breastfeeding rates [48]. Also, early breastfeeding support helped to increase breastfeeding by 24% [49]. In this study, postnatal feeding support was found to be mainly received from health professionals and peers / family through in-person contact in all countries. A systematic review showed that the views and experiences of family members toward breastfeeding support were multi-faceted [50]. Facilitators of exclusive breastfeeding (EBF) were, having good knowledge and skills among healthcare professionals and the support of healthcare services to improve breastfeeding practice [51, 52]. The provision of sufficient information with tailored, practical support is the main reason mothers continue to breastfeed [50, 53].

The COVID-19 pandemic interfered with women obtaining postnatal in-person follow-up care and in-person breastfeeding support [54]. A study that compared the postnatal experiences of women who delivered before and during lockdown in the UK identified a decrease in feeding support from 57% to 40% [36]. Despite the pandemic situation, our study revealed that more than 50% of women received professional support (67% in the pooled sample), ranging from 86% in Thailand to 47% in South Korea. The UK exhibited 57%, a similar rate before lockdown [36]. Meanwhile, a high number of women in South Korea and the UK reported receiving no support.

Health support was found to vary according to the restrictions and pandemic waves in each country. Some countries might face restrictive policies on services, with women receiving virtually no professional and / or peer support. A systematic review of remote support during the COVID-19 pandemic showed that remote breastfeeding support and education combined with support in hospitals reduced the risk of women stopping breastfeeding at three months by 25%, although it is less clear if such an intervention changes the chance of stopping breastfeeding at eight weeks, and three and six months [55]. In this study, 31% of women were found to receive online group support, 33% phone support, and 9% video breastfeeding support. The UK presented similar high rates of online group support (49%) and telephone support (41%) to Thailand (35% and 52%, respectively). Remote support, with online video calls and advice over the telephone, increased during the pandemic because it can help women with infections to self-isolate and receive breastfeeding support due to the COVID-19 control measures [56]. In our study, 59.3% of women who received online support via a video platform reported no difficulties, while 23.5% expressed concern that supporters were unable to clearly see their baby’s latching.

Access to breastfeeding support in hospitals and communities is also restricted due to the control measures in place during the COVID-19 pandemic. Some face-to-face breastfeeding support services by healthcare professionals and breastfeeding peer supporters were temporarily replaced by virtual support via telephone or virtual platforms [57, 58], which may be affected by national COVID-19 infection control measures. Although virtual breastfeeding support has the benefit of convenience, allowing women to receive support at home, a study conducted in the United States of America shows only moderate effectiveness for virtual professional support since it involves certain challenges such as the supporter being unable to assist with latching or analyzing the body language of the baby during the session [58]. How virtual and remote breastfeeding support can best be provided, to whom, and in what circumstances needs further investigation to facilitate the use of technologies for enhancing breastfeeding support.

On the other hand, with no internal support (spouse and family) and external support (professional health services, friends, and employers providing a room for pumping breast milk), women might decide not to continue with breastfeeding [59]. A systematic review shows the importance of community peer support in increasing the duration of exclusive breastfeeding in low- and middle-income countries, especially for infants aged three to six months [60]. Breastfeeding peer support is a good protection strategy since it increases the mother’s self-esteem and confidence [61]. Taiwanese women feel motivated to breastfeed when they have access to services provided by in-center care organizations that facilitate networking with other mothers [62], although during the COVID-19 pandemic, this may have presented a challenge.

### Limitations

The limitations of this study meant that only women who could access the internet could participate and were more likely to be young, have a higher education, and live in urban areas. In addition, recruitment using online nonprobability samples tends to be prone toward sampling participants leading certain lifestyles. Also, in the UK and Brazil, some infant feeding support organizations helped disseminate the online survey information. Such channels might attract women interested in infant feeding (breastfeeding or breastfeeding support) to complete the survey. Thus, the findings of the study cannot be generalized to other populations and settings.

## Conclusion

There are some differences in beliefs toward breastfeeding during the COVID-19 pandemic in Asian countries compared to Brazil and the UK. A positive breastfeeding belief was associated with the practice of infant feeding at the breast. Women from all countries received postpartum infant feeding support from health professionals and peers / family through personal contact. Online group support was higher in Thailand and the UK compared to the remaining countries.

This is the first study to compare different countries and identify important findings on breastfeeding beliefs and feeding practices. In a pandemic situation, governments need to emphasize and disseminate information on the importance of breastfeeding safety, especially in Asian countries. Thus, special effort needs to be placed on lactation support by providing information and strategies to support breastfeeding mothers even during the COVID-19 pandemic. Future studies could explore mothers’ reasons for expressing breast milk instead of breastfeeding at the breast by comparing government support, work status, and support from health professionals.

## Data Availability

All data generated or analyzed during this study are included in this article.

## Abbreviations

BF: Breastfeeding
CDC: Centers for Disease Control and Prevention
EBF: Exclusive breastfeeding
UK: United Kingdom
WHO: World Health Organization
